# N-cadherin antagonism is bronchoprotective in severe asthma models

**DOI:** 10.1126/sciadv.adp8872

**Published:** 2024-11-29

**Authors:** Nicolas L. Pereira, Niccole Schaible, Abhishek Desai, Eunice C. Chan, Ararat J. Ablooglu, Jacqueline Capuano, Erika Lin, Zheming An, Eric Gebski, William Jester, Sundar Ganesan, Nariman Balenga, Cynthia Koziol-White, Reynold A. Panettieri, Sangita Choudhury, Ramaswamy Krishnan, Kirk M. Druey

**Affiliations:** ^1^ Lung and Vascular Inflammation Section, Laboratory of Allergic Diseases, National Institute of Allergy and Infectious Diseases, National Institutes of Health, Bethesda, MD 20892, USA.; ^2^ Center for Vascular Biology Research, Department of Emergency Medicine, Beth Israel Deaconess Medical Center, Boston, MA 02215, USA.; ^3^ Division of Genetics and Genomics, Manton Center for Cell Discovery Research, Department of Pediatrics, Boston Children’s Hospital, Harvard Medical School, Boston, MA 02115, USA.; ^4^ Rutgers Institute for Translational Medicine and Science, Child Health Institute of New Jersey, Rutgers, the State University of New Jersey, New Brunswick, NJ 08901, USA.; ^5^ Biological Imaging Section, National Institute of Allergy and Infectious Diseases, National Institutes of Health, Bethesda, MD 20892, USA.

## Abstract

Severe asthma induces substantial mortality and chronic disability due to intractable airway obstruction, which may become resistant to currently available therapies including corticosteroids and β-adrenergic agonist bronchodilators. A key effector of these changes is exaggerated airway smooth muscle (ASM) cell contraction to spasmogens. No drugs in clinical use effectively prevent ASM hyperresponsiveness in asthma across all severities. We find that N-cadherin, a membrane cell-cell adhesion protein up-regulated in ASM from patients with severe asthma, is required for the development of airway obstruction induced by allergic airway inflammation in mice. Inhibition of N-cadherin by ADH-1 reduced airway hyperresponsiveness independent of allergic inflammation, prevented bronchoconstriction, and actively promoted bronchodilation of airways ex vivo. ADH-1 inhibited ASM contraction by disrupting N-cadherin–δ-catenin interactions, which decreased intracellular actin remodeling. These data provide evidence for an intercellular communication pathway mediating ASM contraction and identify N-cadherin as a potential therapeutic target for inhibiting bronchoconstriction in asthma.

## Teaser

N-cadherin inhibition prevents airway obstruction in allergic asthma models and elicits bronchodilation.

## INTRODUCTION

Asthma is a chronic inflammatory lung disease that affects more than 300 million people worldwide (*1*). Episodic and reversible airway obstruction evokes shortness of breath, wheezing, and cough, which results from exaggerated bronchoconstriction to airway spasmogens, termed “airway hyperresponsiveness (AHR)”. This phenotype encompasses both increased maximal responses and heightened reactivity to submaximal concentrations of contractile agonists present in the airways including acetylcholine (ACh), histamine, and leukotrienes and indirect physical stimuli such as exercise or cold exposure (*2*). Although AHR is a universal feature of asthma, its underpinnings are probably heterogeneous and complex (*3*, *4*).

Soluble airway mediators associated with type 2 lung inflammation elicit lung remodeling that includes epithelial damage, increased deposition of extracellular matrix (ECM) components around the airway wall, and airway smooth muscle (ASM) cell hypertrophy and hyperplasia, the extent of which correlate with disease severity and clinical outcomes (*5*). These physical changes increase airway thickness and stiffness, exerting direct and indirect effects on ASM contractility (*6*, *7*). Type 2 cytokines interleukin-4 (IL-4) and IL-13 enhance ASM contraction (*8*). However, uncontrolled AHR may be present even in patients with minimal airway inflammation (*9*), suggesting that intrinsic dysregulation of lung structural components, including ASM cells, contributes to AHR in certain contexts (*10*).

Studies of the biomechanical properties of ASM from patients with asthma have been confounded by anatomical and methodological limitations. Early studies were focused on tracheal ASM strips or cultured tracheal-derived ASM cells. These experiments yielded conflicting results as to whether differences in contractility exist between healthy and asthma-derived samples (*11*, *12*). Imaging and ventilation measurements done in patients with active bronchoconstriction have localized the smaller peripheral bronchioles as the predominant sites of airway narrowing in asthma (*13*, *14*), suggesting the importance of studying ASM from intrapulmonary airways. More recent examination of intrapulmonary muscle strips demonstrated increased contractile force and stiffness in asthma-derived samples compared to controls in response to methacholine (MCh, an ACh analog) without concordant impairment in relaxation responses (*15*). Proteomic studies showed up-regulated cytoskeletal and contraction-related smooth muscle specific proteins in lung tissue from patients with asthma including zyxin, myosin light chain (MLC) kinase, and smoothelin compared to controls.

Despite the identification of these and other potential therapeutic targets, few drugs currently exist to limit ASM contraction in asthma. Although the development of anti-inflammatory biologics targeting asthma-associated cytokines has transformed the care of patients with chronic asthma over the last decade (*16*), these agents have no defined role in acute bronchoconstriction. ASM-targeted drugs in clinical use promote bronchodilation (e.g., β-adrenergic agonists), and these are prone to tolerance and lack of efficacy after repeated use, which can increase mortality and morbidity (*17*).

Cadherins are a family of widely expressed integral membrane proteins whose Ca^2+^-dependent homotypic interactions between adjacent cells mediate intercellular adhesion. In asthma, allergens elicit T2 airway inflammation by disrupting epithelial (E)–cadherin–dependent adherens junctions in respiratory epithelial cells (*18*, *19*). ASM cells primarily express neuronal (N)–cadherin but not E-cadherin. While global *Cdh2* knockout in mice is embryonic lethal, tissue-specific deletion has revealed important functions of N-cadherin in muscle tissues (*20*). Mice with inducible N-cadherin deficiency in adult myocardium (*Cdh2^fl/fl^-*α*MHC-MerCre*) are prone to developing cardiomyopathy and arrhythmias associated with impaired adhesion between cardiomyocytes (*21*). The role of N-cadherin in AHR is unknown.

We set out to determine the importance of N-cadherin for ASM contraction and discovered that N-cadherin is essential for the development of AHR in allergen-challenged mice and contractile force development in human ASM (HASM) cells through its effects on collective force transmission and actin remodeling. Pharmacological N-cadherin antagonism is bronchoprotective and elicits bronchodilation, even in airways desensitized to β-agonists, highlighting a previously unrecognized therapeutic approach to AHR in severe asthma.

## RESULTS

### N-cadherin is expressed in ASM cells

**Fig. 1.**
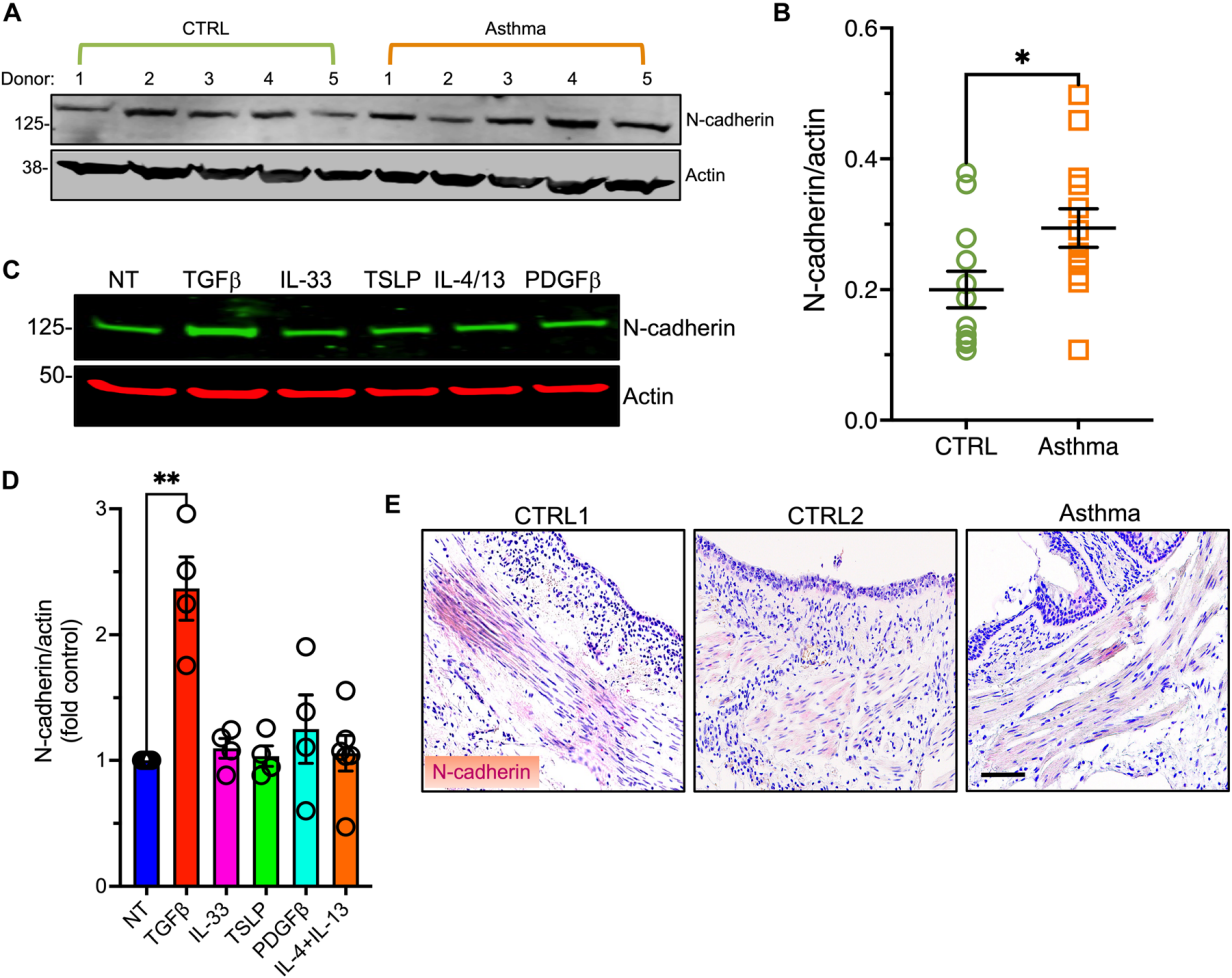
N-cadherin expression in ASM. (**A**) N-cadherin detected in HASM lysates by immunoblotting (*n* = 5 donors per group). (**B**) N-cadherin/β-actin ratios; means ± SEM from *n* = 12 to 13 donors per group, *P = 0.03, unpaired *t* test. (**C**) N-cadherin in lysates from serum starved HASM cells left untreated or stimulated with TGFβ (5 ng/ml), IL-33 (50 ng/ml), TSLP (10 ng/ml), PDGFβ (10 ng/ml), or IL-4 plus IL-13 (100 ng/ml each) for 16 hours. (**D**) N-cadherin/actin ratios. Means ± SEM from *n* = 4 to 6 sets of cells. **P = 0.002 versus control, Kruskal-Wallis ANOVA, Dunn multiple comparisons. (**E**) Human lung sections immunostained with N-cadherin antibody (dark red) and counterstained with hematoxylin. Scale bar, 100 μm. Images represent *n* = 13 airways from 9 donors.

We detected N-cadherin protein in lysates of HASM cells by immunoblotting (Fig. 1A). N-cadherin expression was higher in lysates of ASM cells cultured postmortem from the lungs of patients who died of acute asthma compared to ASM cells from healthy controls (Fig. 1B). The demographic information about these subjects and other relevant information are shown in table S1. To determine the mechanisms underlying these differences, we treated HASM cells with asthma-related cytokines, several of which [IL-4/13 and transforming growth factor–β (TGFβ)] are known to directly increase ASM contractility (*8*, *22*). TGFβ, but not IL-33, thymic stromal lymphopoietin (TSLP), IL-4 plus IL-13, or platelet-derived growth factor–β (PDGFβ) increased N-cadherin expression in HASM lysates (Fig. 1, C and D). We assessed N-cadherin expression in human lung sections using immunohistochemistry. N-cadherin immunostaining was confined to the ASM bundles surrounding airways (Fig. 1E), with the strongest signal in airways 1 to 3 mm in diameter, consistent with terminal (conducting) bronchi and pre-preterminal bronchioles (*23*). There was fainter but detectable N-cadherin immunostaining in some terminal bronchioles (<1 mm in diameter). The mean (±SEM) diameter of N-cadherin^+^ airways was 1.13 ± 0.3 mm.

### N-cadherin is required for the development of AHR in experimental asthma in mice

**Fig. 2.**
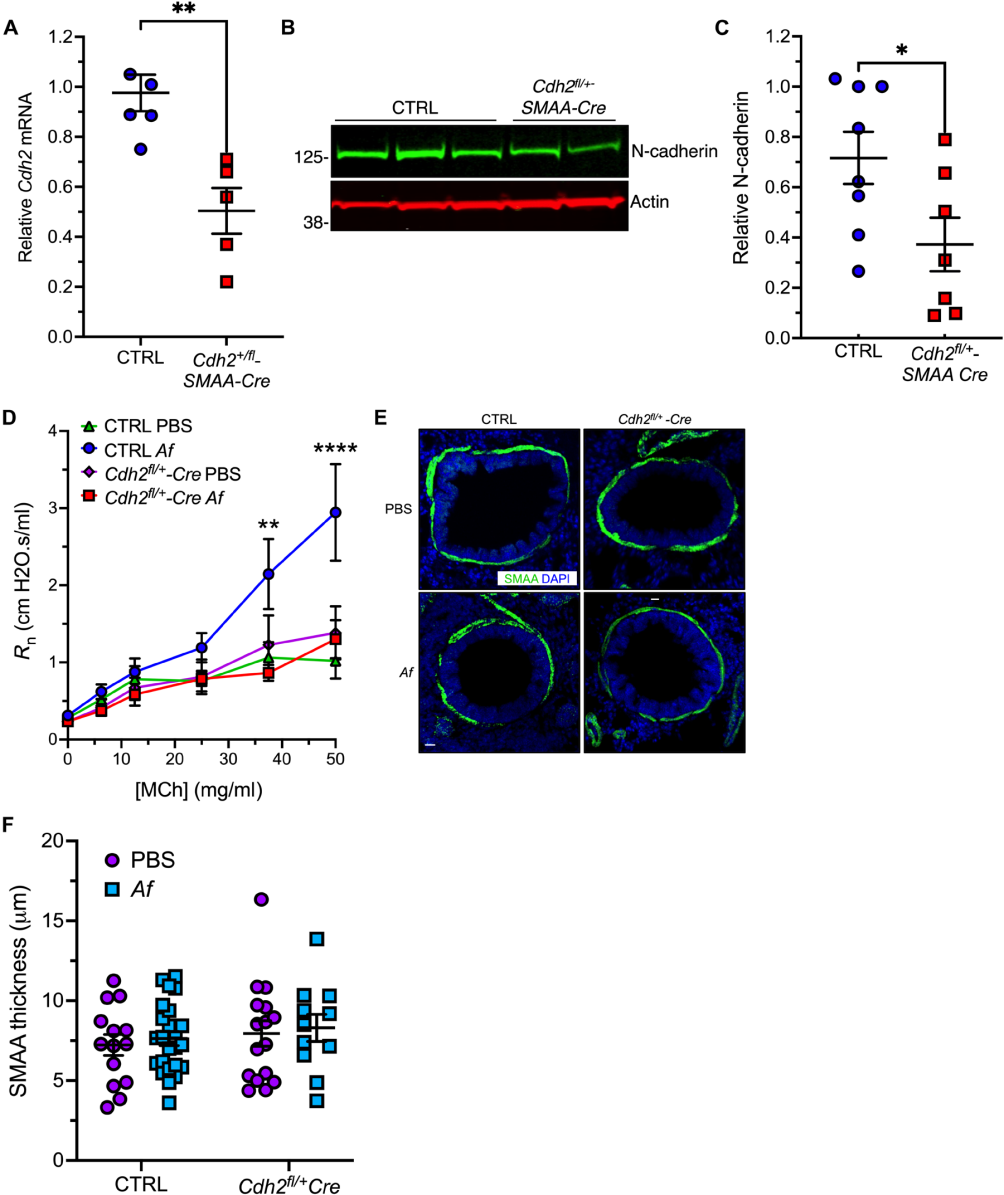
ASM-derived N-cadherin is required for allergen-induced AHR. (**A**) Relative *Cdh2/Actb* expression in lung RNA from control (CTRL = *Cdh2^fl/+^*or *Cdh2^fl/fl^*) and *Cdh2*^*fl*/+^-*SMAA Cre* mice. Means ± SEM of *n* = 5 to 6 mice per group. **P = 0.002, unpaired *t* test. (**B** and **C**) N-cadherin protein expression in lung lysates from control or *Cdh2*^*fl*/+^-SMAA Cre mice detected by immunoblotting (B) and N-cadherin/β-actin ratios determined by ImageJ analysis (C). Means ± SEM from *n* = 7 to 8 mice per group. *P = 0.03, unpaired *t* test. (**D**) Newtonian lung resistance (*R*_n_) in naïve or *Af*-challenged mice. Means ± SEM from *n* = 7 to 10 mice per group. **P = 0.003 and ****P < 0.0001 (CTRL versus *Cdh2^fl/+^-SMAA Cre*, *Af*-treated), two-way ANOVA, Sidak multiple comparisons. (**E** and **F**) Smooth muscle thickness around airways [delineated by staining with 4′,6-diamidino-2-phenylindole (DAPI), blue] detected by immunostaining with smooth muscle α-actin antibody (SMAA, green) and quantified by ImageJ (F). Means ± SEM from *n* = 14 to 24 airways per group. Scale bars, 20 μm.

To study the impact of N-cadherin deficiency on bronchial smooth muscle functions in vivo, we crossed *Cdh2^fl/fl^* mice with mice expressing Cre recombinase driven by the α-smooth muscle actin (SMAA) promoter (*24*). We could not generate homozygous *Cdh2^fl/fl^-SMAA Cre* mice, suggesting that smooth muscle N-cadherin expression is required for proper embryonic development. However, *Cdh2* mRNA abundance analyzed by quantitative PCR (Fig. 2A) and N-cadherin protein expression (Fig. 2, B and C) in lysates from lung tissue assessed by immunoblotting were reduced by ~50% in *Cdh2^fl/+^-SMAA Cre* mice compared to controls (*Cdh2^fl/fl^* or *Cdh2^fl/+^*). These heterozygous smooth muscle-specific N-cadherin “knockdown” mice were viable and fertile, with no gross phenotypic abnormalities at baseline.

To induce AHR, we sensitized mice with allergen extracts of *Aspergillus fumigatus* (*Af*), a ubiquitous environmental mold associated with severe asthma (*25*), followed by consecutive respiratory challenges with phosphate-buffered saline (PBS) or *Af* (see schematic in fig. S1). We measured Newtonian resistance (*R*_n_), which represents the caliber of conducting airways (*26*) in live animals by invasive plethysmography at baseline and in response to various doses of MCh. Although baseline *R*_n_ was comparable in N-cadherin knockdown mice and controls, *Af*-challenged *Cdh2^fl/+^-SMAA Cre* mice failed to develop AHR, with lung *R*_n_ values that were comparable to those in mice of either genotype challenged with PBS alone (Fig. 2D).

We also evaluated respiratory system resistance (*R*_rs_), a parameter affected by lung parenchymal tissue elasticity and chest wall compliance (*27*). *R*_rs_ values typically have a wider range than *R*_n_, and subtle differences in baseline *R*_rs_ have been described in naïve mice (*28*). *R*_rs_ was significantly lower in *Cdh2^fl/+^-Cre* mice than in controls in the presence or absence of allergen challenge (fig. S2). To determine whether these changes result from altered airway smooth muscle mass, we immunostained lung sections with smooth muscle α actin antibody, a marker of ASM cells. The SMAA^+^ area around bronchioles was equivalent in mice of either genotype, irrespective of allergen challenge (Fig. 2, E and F). These findings suggest that the lower *R*_rs_ values and near absence of AHR in *Cdh2^fl/+^-SMAA Cre* was not due to a defect in bronchial smooth muscle development or airway remodeling.

### Smooth muscle N-cadherin deficiency has little effect on type 2 lung inflammation

**Fig. 3.**
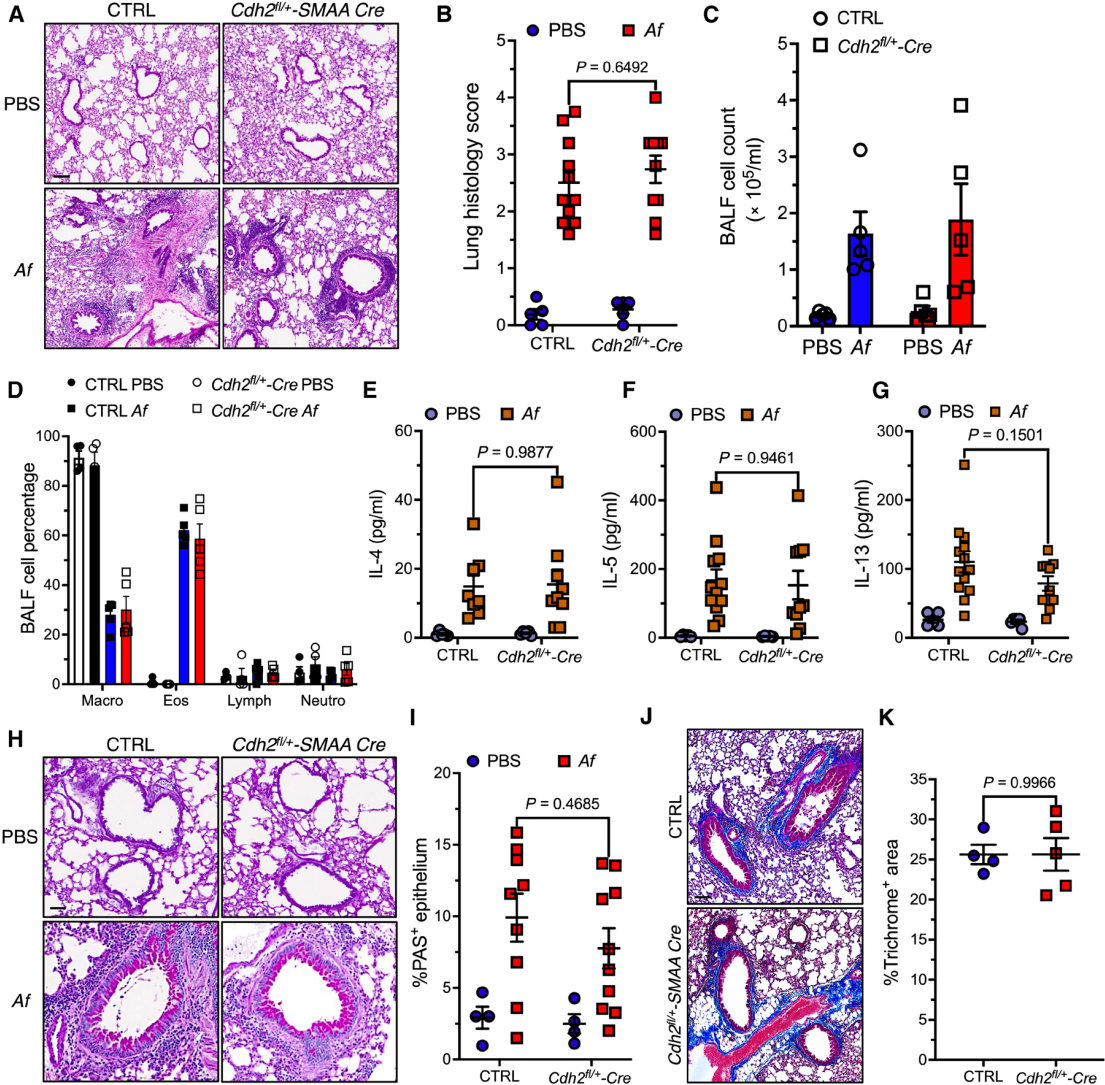
Smooth muscle specific *Cdh2* haploinsufficiency does not affect allergic lung inflammation or airway remodeling. (**A** and **B**) Representative images (A) of hematoxylin/eosin-stained lung sections from mice and lung histology score (B). Means ± SEM of *n* = 5 to 11 mice per group. *P* values were determined by two-way ANOVA with Sidak multiple comparisons. (**C** and **D**) BALF total leukocyte counts (C) and differential composition (D) in PBS or *Af*-challenged mice. Means ± SEM from *n* = 4 to 5 mice per group. (**E** to **G**) Allergy-associated cytokines IL-4 (E), IL-5 (F), or IL-13 (G) in BALF. Means ± SEM from 5 to 13 mice per group; *P* values were determined by two-way ANOVA with Tukey multiple comparisons. (**H** and **I**) Representative images (H) of PAS-stained lung sections from mice and percent PAS^+^ epithelial area (I). Means ± SEM from *n* = 4 to 10 mice per group. *P* values were determined by two-way ANOVA with Sidak multiple comparisons. (**J** and **K**) Representative images (J) of Masson-Trichrome–stained lung sections from mice and Trichrome^+^ area/airway circumference (K). Means ± SEM from *n* = 4 to 5 mice per group. *P* value was determined by unpaired *t* test. Scale bars, 100 μm.

To determine the mechanism(s) underlying the absence of AHR in allergen-challenged N-cadherin knockdown mice, we first examined various parameters of lung inflammation. We evaluated peribronchial and perivascular inflammation in hematoxylin/eosin-stained lung sections using a published scoring system (*29*). Lungs from PBS-challenged *Cdh2^fl/+^-SMAA Cre* mice appeared normal (Fig. 3A). Peribronchial and perivascular accumulation of leukocytes (predominantly eosinophils) in *Af*-challenged *Cdh2^fl/+^-SMAA-Cre* mice was equivalent to that in *Af*-challenged control mice (Fig. 3B). Likewise, total leukocyte counts (Fig. 3C), leukocyte composition (Fig. 3D), and levels of canonical T2 (IL-4, IL-5, and IL-13) (Fig. 3, E to G) or other asthma-related cytokines (IL-1β, tumor necrosis factor–α, IL-9, IL-10, IL-17A, and eotaxin) (fig. S3, A to F) were similar in bronchoalveolar lavage fluid (BALF) from *Af*-challenged control and *Cdh2^fl/+^-SMAA Cre* mice. Among several mechanisms, T2 cytokines promote airway obstruction by increasing mucin expression in bronchial epithelial cells (*30*). We assessed mucin expression within the respiratory epithelium by periodic acid–Schiff (PAS) staining (Fig. 3H). The percentage of PAS staining in the airway epithelium was equivalent *Cdh2^fl/+^-SMAA Cre* and control mice (Fig. 3I). Epithelial damage and allergic cytokines also induce subepithelial deposition of ECM proteins like collagen (*5*). We assessed bronchiolar collagen content in lung sections by Masson Trichrome staining. The Trichrome^+^ airway area was qualitatively and quantitatively similar in *Cdh2^fl/+^-SMAA Cre* and control mice (Fig. 3, J and K). Overall, these findings suggested that N-cadherin–deficient mice were resistant to developing AHR independent of changes in allergic airway inflammation or remodeling.

### N-cadherin antagonism perturbs airway biomechanics

**Fig. 4.**
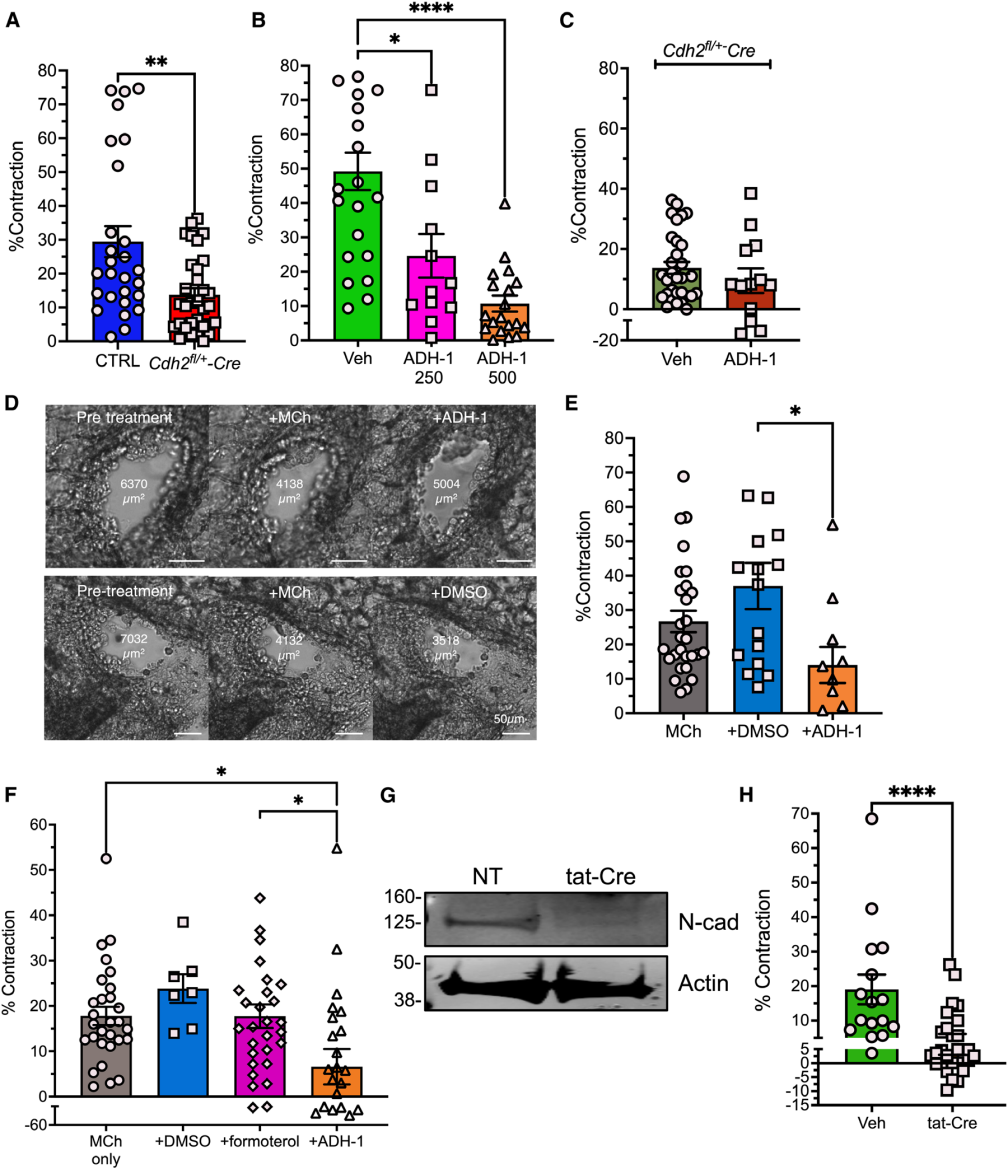
N-cadherin disruption is bronchoprotective and bronchodilatory. (**A** to **C**) Percentage contraction (from baseline area) in PCLS from *Cdh2^fl/+^-SMAA Cre* mice (A) or pretreated with vehicle or ADH-1 [μg/ml] [(B) and (C)] and stimulated with MCh (10 μM, 20 min). (A) Means ± SEM from *n* = 27 to 32 airways per group; **P = 0.005, Mann-Whitney. (B) Means ± SEM from *n* = 12 to 22 airways per group; *P = 0.01 and ****P < 0.0001 versus control, one-way ANOVA, Dunnett multiple comparisons. (C) Means ± SEM from *n* = 12 to 34 airways per group. (**D** and **E**) PCLS contracted with MCh (10 μM, 20 min) and then treated with vehicle (0.5% DMSO) or ADH-1 (500 μg/ml) for an additional 10 min. Means ± SEM from *n* = 11 to 29 airways per group *P = 0.01 versus MCh, one-way ANOVA, Tukey multiple comparisons. (**F**) PCLS were left untreated or treated with formoterol (100 ng/ml × 20 min). Airways were then treated with MCh (10 μM, 20 min), followed by addition of either vehicle (DMSO), formoterol, or ADH-1 (500 μg/ml). Means ± SEM from *n* = 7 to 30 airways per group; *P = 0.01, one-way ANOVA, Tukey multiple comparisons. (**G**) Lysates from PCLS treated with tat-Cre (5 μM) or vehicle alone for 72 hours immunoblotted with N-cadherin and β-actin antibodies. (**H**) MCh-induced airway contraction in vehicle-treated or tat-Cre–treated PCLS. Means ± SEM from *n* = 16 to 28 airways per group; ****P < 0.0001, Mann Whitney.

Lung N-cadherin expression is strongest within the ASM bundle, and the salutary effects of N-cadherin deficiency on AHR in the acute allergen challenge model are largely independent of lung structural changes. Therefore, we hypothesized that N-cadherin promotes bronchial contraction. To test this hypothesis, we examined airway narrowing in precision cut lung slices (PCLS). Although baseline airway luminal area was similar in PCLS from naïve *Cdh2^fl/+^-SMAA Cre* and control mice (fig. S4), the airways from N-cadherin knockdown mice contracted significantly less in response to MCh than those from controls (Fig. 4A). To determine the therapeutic applications of this finding, we evaluated contraction of murine PCLS treated with a clinically relevant N-cadherin antagonist. ADH-1 (N-Ac-CHAVC-NH2) is a cyclic peptide that contains an evolutionarily conserved His-Ala-Val sequence present in the first extracellular domain of N-cadherin. This sequence is thought to be essential for N-cadherin homotypic binding, permitting ADH-1 to act as a competitive antagonist of N-cadherin–mediated intercellular adhesion (*31*). ADH-1 treatment of PCLS did not affect baseline airway diameter (fig. S4) but significantly prevented MCh-induced contraction in a dose-dependent fashion (Fig. 4B). ADH-1 had little impact on MCh-induced contraction in PCLS from *Cdh2^fl+^-SMAA Cre* mice, confirming its on-target activity (Fig. 4C). Moreover, application of ADH-1 to MCh-preconstricted PCLS elicited bronchodilation (Fig. 4, D and E). ADH-1 induced airway relaxation even in airways previously treated with and desensitized (i.e., unresponsive) to repeated doses of β-agonist (formoterol) (Fig. 4F). These results suggest that N-cadherin antagonism has both bronchoprotective and bronchodilatory benefits.

Heterozygous gene knockout mice may display compensatory effects. To address this potential confounding factor, we first analyzed contraction of PCLS from adult *Cdh2^fl/+^*mice treated with cell permeable tat-Cre recombinase to induce acute *Cdh2* deletion (*32*). N-cadherin expression was substantially reduced in lysates from tat-Cre–treated PCLS by immunoblotting (Fig. 4G). Airways in slices incubated with tat-Cre contracted significantly less in response to MCh than did those treated with vehicle alone (Fig. 4H).

To further explore the mechanisms underlying N-cadherin’s regulation of AHR, we conducted RNA sequencing of lung tissue collected from *Af-*challenged *Cdh2^fl/+^*-*Cre* and control mice. This analysis revealed only 20 genes (including *Cdh2*) that that were significantly differentially expressed (full list in data file S1), and only three of these transcripts (*Cyp1a1*, *Cfd*, and *Xirp1*) exhibited a >2-fold change in expression (fig. S5A). A limited number of categories were enriched in the geneset including immune system phenotype and abnormal protein levels (fig. S5B).

### N-cadherin promotes HASM contractility

Our cumulative data suggest that N-cadherin promotes the development of AHR, not through extensive transcriptional changes or lung remodeling, but rather by means of increased ASM contraction. To test this hypothesis, we cultured HASM cells upon deformable substrates containing pre-embedded fluorescent beads and then performed traction force microscopy. This method is based on analysis of bright-field cellular images and their corresponding traction maps (*33*, *34*). From each map, we calculated the strain energy (i.e., the energy that is imparted to the substrate by the contracting cells (in pJ), which represents the total cellular contraction, and the probability distribution function of the traction data, which represents ASM force distribution. We chose histamine over MCh because it elicits more robust responses in unmanipulated primary HASM cells, which are characterized by poor m3 muscarinic receptor expression.

**Fig. 5.**
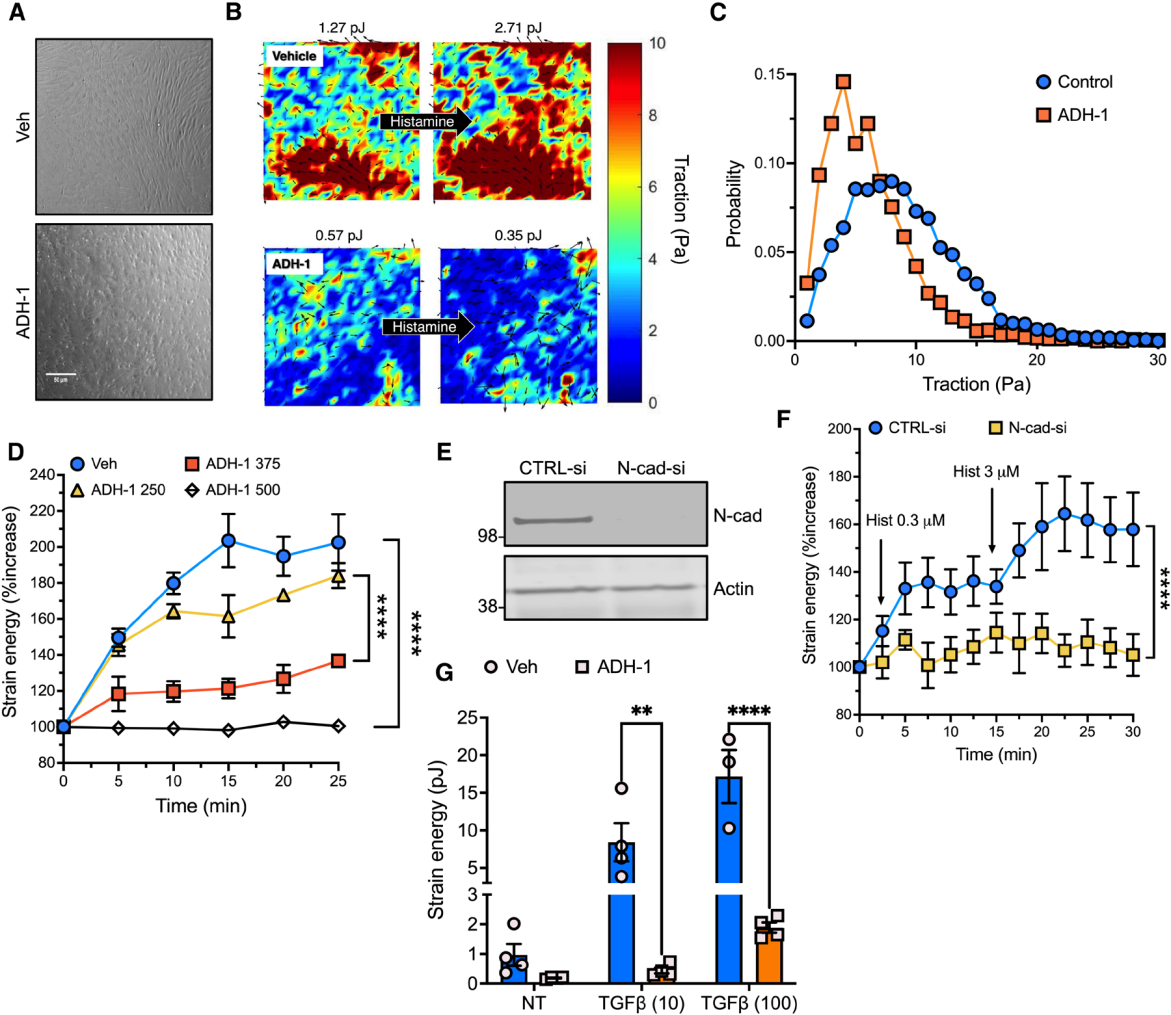
N-cadherin antagonism or deficiency impairs HASM contraction. (**A** and **B**) Representative cellular images (A) and corresponding traction maps (B) in HASM cells pretreated with vehicle (Veh) or ADH-1 (500 μg/ml) for 24 hours and then stimulated with histamine (3 μM, 20 min). Scale bar, 50 μm. Total traction per field of view (represented as the strain energy in pJ) noted above each field. (**C**) Probability distribution of traction and its variance in Veh-treated or ADH-1–treated HASM cells. Each distribution was calculated over *n* = 4 distinct fields comprising ~400 cells per field in two independent experiments. (**D**) Time course of histamine-induced contraction in HASM treated with various doses of ADH-1 (μg/ml). Means ± SEM from *n* = 3 to 4 distinct fields comprising ~400 cells per field. ****P < 0.0001 vs. control, two-way ANOVA, Tukey multiple comparisons. (**E**) Lysates from siRNA-treated HASM cells immunoblotted with N-cadherin or actin antibody, representative of *n* = 4 independent experiments. (**F**) Traction in siRNA-treated HASM cells stimulated sequentially with increasing concentrations of histamine at the indicated times (arrows). Means ± SEM from *n* = 8 sets of cells per group. ****P < 0.0001, two-way ANOVA, Sidak multiple comparisons. (**G**) Traction in HASM cells treated with TGFβ (ng/ml) for 18 hours in the presence or absence of ADH-1 (500 μg/ml). Means ± SEM from *n* = 3 to 4 distinct fields per group. **P = 0.005 and ****P < 0.0001, two-way ANOVA, Sidak multiple comparisons.

Pretreatment of HASM with ADH-1 reduced the ASM contractile force at homeostasis, both in terms of magnitude (Fig. 5 A and B) and variance in the traction distribution (Fig. 5C). Moreover, ADH-1 time and dose-dependently inhibited histamine-induced contraction (Fig. 5D). ADH-1 treatment did not affect cell viability even at high doses, as reflected by intracellular ATP levels that were equivalent to vehicle [dimethyl sulfoxide (DMSO)]–treated cells (fig. S6A). We confirmed these results using a multiplexed assay that assesses cell viability, cytotoxicity, and apoptosis. The activity of a ubiquitous intracellular protease on a cell-permeable peptide substrate generates a fluorescent signal proportional to the number of live cells. Cytotoxicity is evaluated by measuring fluorescence produced by digestion of a cell impermeable peptide by a protease released by dead cells. Next, addition of a luminogenic substrate of caspase 3/7 to cell lysates is used to evaluate apoptosis. By these measures, treatment of HASM cells with ADH-1 did not affect viability (fig. S6B), induce cytotoxicity (fig. S6C), or elicit apoptosis (fig. S6D).

To corroborate the effects of ADH-1 on ASM contractility, we depleted N-cadherin in HASM cells acutely using RNA interference. Treatment with *Cdh2*-targeted siRNAs nearly abolished N-cadherin expression compared to transfection with a nontargeting control siRNA (Fig. 5E). N-cadherin–deficient HASM cells contracted significantly less in response to histamine than control cells did (Fig. 5F).

Moreover, we hypothesized that N-cadherin–dependent mechanotransduction mediates some outcomes of TGFβ signaling in ASM, more specifically, increased contractility (*22*, *35*). To test this hypothesis, we treated HASM cells with TGFβ in the presence or absence of ADH-1 and performed traction force microscopy. TGFβ treatment increased traction compared to vehicle alone, while ADH-1 pretreatment strongly blocked this response (Fig. 5G). These results suggest that TGFβ-induced up-regulation of N-cadherin represents one mechanism by which this cytokine augments ASM contraction.

### N-cadherin antagonism reduces actin remodeling in HASM

To determine the mechanism(s) by which N-cadherin regulates ASM contraction, we first examined several canonical excitation-contraction signaling pathways. Spasmogens such as histamine, ACh [and its analogs MCh or carbachol (CCh)] activate heterotrimeric G proteins through G protein–coupled receptors to exchange guanosine triphosphate (GTP) for guanosine diphosphate. Activated G protein α subunits of the q/11 subfamily stimulate several effectors such as phospholipase Cβ, which in turn promotes release of Ca^2+^ from intracellular stores. Ca^2+^ bound to calmodulin activates MLC kinase, culminating in actin-myosin cross-bridging and cell shortening (*36*). Unexpectedly, ADH-1 pretreatment of HASM cells had no effect on these pathways, including intracellular Ca^2+^ flux (fig. S7, A and B), MLC phosphorylation, or MLC phosphatase (MYPT1) phosphorylation in response to histamine (fig. S7, C to E) or CCh (fig. S7F).

**Fig. 6.**
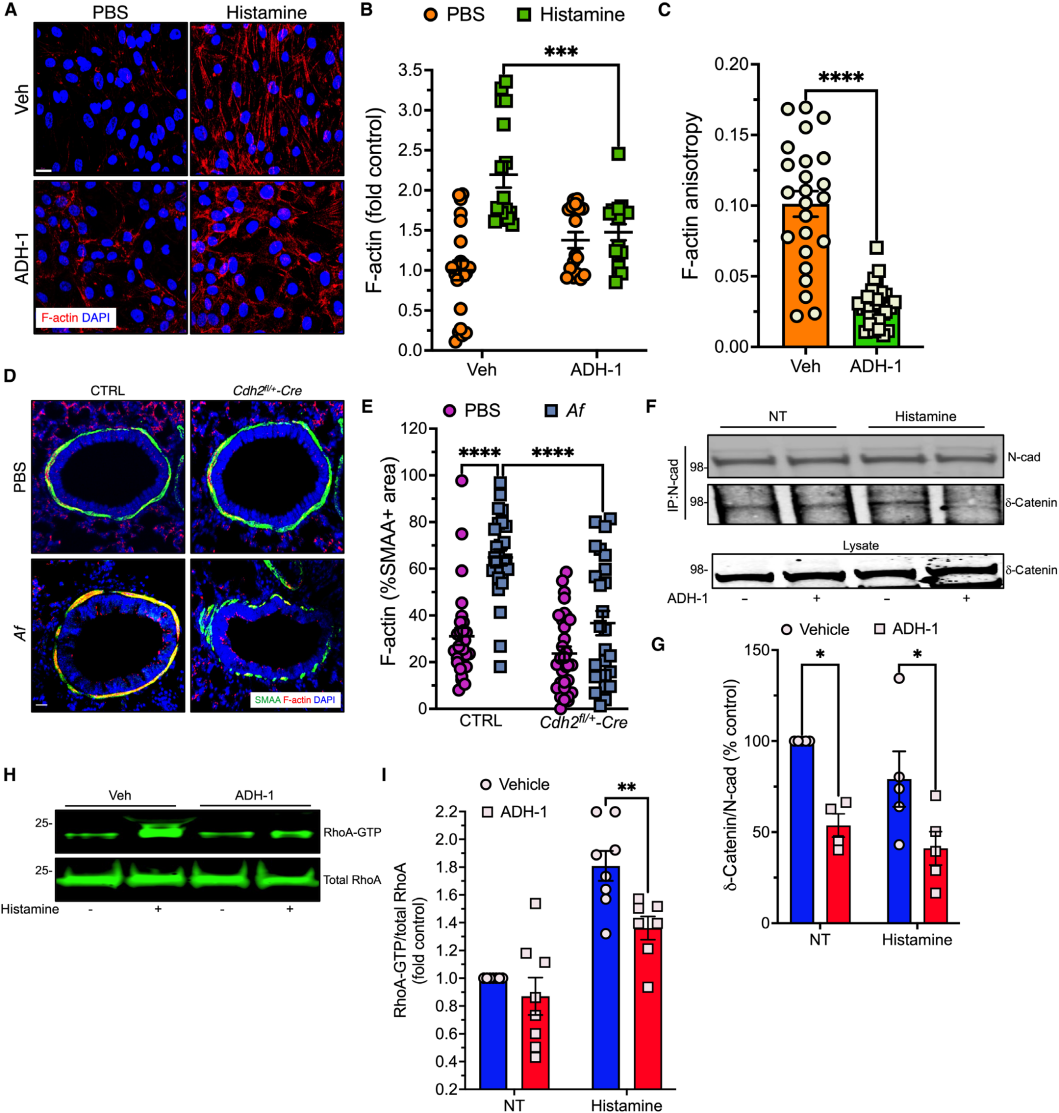
ADH-1 impairs agonist-induced actin remodeling in HASM by inhibiting RhoA activation. (**A**) HASM cells pretreated with vehicle or ADH-1 (250 μg/ml) and left untreated or stimulated with histamine (10 μM) for 10 min, fixed and stained with phalloidin (red) and DAPI (blue). Scale bar, 20 μm. (**B** and **C**) Total F-actin (B) and F-actin anisotropy (C) quantified; means ± SEM from *n* = 17 to 22 separate fields per group. ***P = 0.0004, two-way ANOVA, Sidak multiple comparisons (B); ****P < 0.0001, Mann-Whitney (C). (**D**) Lung sections from *Cdh2^fl/+^-*SMAA Cre or control mice challenged with PBS or *Af* stained with phalloidin (red), SMAA (green), and DAPI (blue). (**E**) F-actin in bronchial smooth muscle; means ± SEM from *n* = 28 to 31 airways per group. ****P < 0.0001, two-way ANOVA, Tukey multiple comparisons. Scale bar, 20 μm. (**F**) N-cadherin immunoprecipitates and total lysates from HASM cells treated as indicated and immunoblotted with N-cadherin or δ-catenin antibody. (**G**) δ-catenin/N-cadherin ratios. Means ± SEM of from *n* = 4 to 5 independent experiments. *P = 0.03, two-way ANOVA, Sidak multiple comparisons. (**H**) RhoA-GTP and total RhoA in HASM cell lysates detected with RhoA antibody. (**I**) Active RhoA in cell lysates treated as indicated. Means ± SEM of from *n* = 6 independent experiments. **P = 0.008, two-way ANOVA, Sidak multiple comparisons.

Apart from the pathways mediating ASM cell shortening, remodeling of the actin cytoskeleton transmits force by increasing overall cell rigidity (*37*). ASM mechanotransduction elicits formation of discrete submembranous foci containing actin regulatory and signaling proteins called adhesomes. These hubs promote formation of parallel F-actin stress fibers oriented along the cell’s longitudinal axis, which transmit tension generated by cell shortening to the cell membrane. We assessed F-actin formation by staining with fluorophore-conjugated phalloidin. N-cadherin blockade with ADH-1 markedly reduced histamine-induced actin remodeling in terms of overall F-actin quantities (Fig. 6, A and B) and the alignment of stress fibers (anisotropy) (Fig. 6C). To determine the in vivo implications of these in vitro findings, we stained lung sections with phalloidin and SMAA antibody to evaluate actin polymerization in bronchial smooth muscle in situ. Consistent with the findings in HASM cells, F-actin staining was significantly reduced in bronchial smooth muscle of allergen-challenged *Cdh2^fl/+^-SMAA Cre* mice compared to controls (Fig. 6, D and E).

We next examined the molecular mechanisms by which N-cadherin regulates actin remodeling. N-cadherin connects to the actin cytoskeleton through several linker proteins including β-catenin, δ-catenin, and α-catenin (which directly binds actin filaments) (*38*). Because β-catenin is not directly involved in actin polymerization (*39*, *40*), we focused further attention on δ-catenin, a scaffold protein that regulates the stability of cadherin-catenin complexes in various cell types but has not yet been studied in the context of muscle contraction. Specifically, δ-catenin dissociated from cadherin complexes positively or negatively regulates activity of the small GTPase RhoA, a key modulator of actin polymerization, depending on the cell type (*41*). We find that ADH-1 treatment of HASM cells was associated with reduced amounts of δ-catenin in lysates immunoprecipitated with N-cadherin antibody (Fig. 6, F and G). Moreover, ADH-1 pretreatment significantly reduced histamine-induced RhoA activation (Fig. 6, H and I). These cumulative results suggest that N-cadherin–δ-catenin–mediated mechanosignaling in ASM elicits actin remodeling by activating RhoA.

### ADH-1 alleviates AHR in experimental asthma in mice

**Fig. 7.**
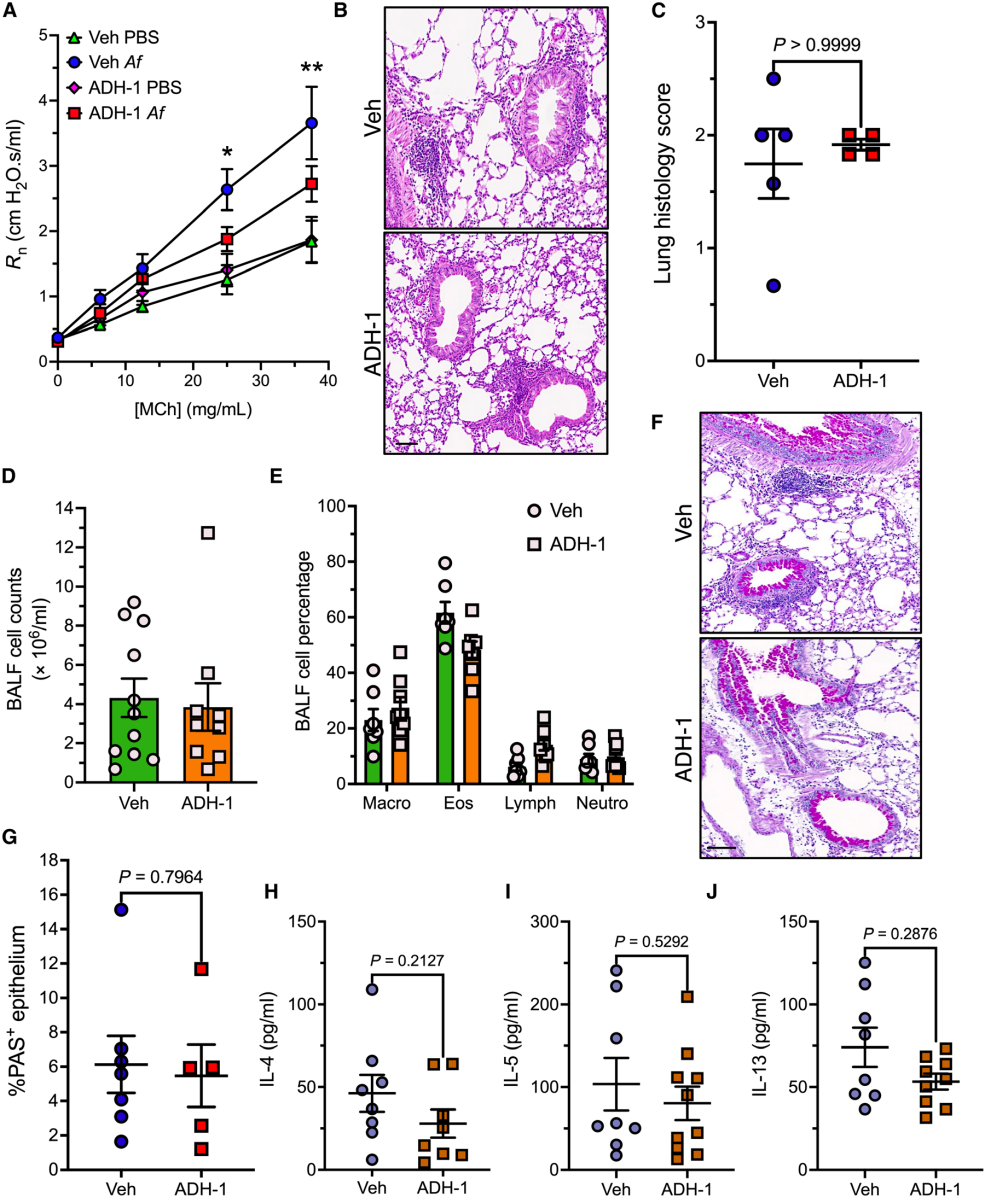
ADH-1 reduces allergen induced AHR independent of allergic inflammation. (**A**) Lung resistance in PBS- or *Af*-challenged mice pretreated with vehicle (Veh) or ADH-1 (200 mg/kg via intraperitoneal injection). Means ± SEM from *n* = 11 to 15 mice per group. *P = 0.01 and **P = 0.005, two-way ANOVA, Benjamini-, Krieger-, and Yekutieli-corrected multiple comparisons. (**B** and **C**) Representative hematoxylin/eosin-stained lung sections (B) and lung histology score (C). Means ± SEM from *n* = 4 to 5 mice per group. *P* value was determined by Mann Whitney *U* test. (**D** and **E**) BALF total leukocyte counts (D) and differential composition (E) in *Af-*challenged mice pretreated with Veh or ADH-1. Means ± SEM from *n* = 4 to 5 mice per group. (**F** and **G**) Representative PAS-stained lung sections (F) and percent PAS^+^ epithelial area (G). Means ± SEM from *n* = 5 to 7 mice per group. *P* value was determined by unpaired *t* test. (**H** to **J**) Levels of IL-4 (H), IL-5 (I), or IL-13 (J) in BALF from *Af*-challenged mice. Means ± SEM from 8 to 10 mice per group, *P* values shown were determined by two-way ANOVA with Tukey multiple comparisons. Scale bars, 100 μm.

To evaluate the potential of ADH-1 as a treatment for asthma, we administered ADH-1 to mice after *Af* sensitization but before measurements of lung resistance (fig. S8). Like the genetic model, pretreatment with ADH-1 significantly reduced MCh-induced airway obstruction in *Af*-challenged Balb/c mice (Fig. 7A). ADH-1 treatment had no appreciable impact on inflammation including peribronchial and perivascular inflammation as assessed by histology (Fig. 7, B and C), total leukocyte numbers (Fig. 7D), or leukocyte composition (Fig. 7E) in BALF. Likewise, epithelial mucin expression assessed by PAS staining (Fig. 7, F and G) or asthma-related cytokines in BALF (Fig. 7, H and J, and fig. S9, A to F) were equivalent in vehicle-treated or ADH-1–treated mice.

## DISCUSSION

AHR presents an intractable problem for the treatment of patients with acute severe asthma symptoms as it may be associated with reduced responsiveness to bronchoconstrictors, and thus resistant to conventional bronchodilators (*42*–*44*). The efficacy of β2 agonist bronchodilators to relax ASM is also adversely impacted by β2 adrenergic receptor desensitization and a complex signaling pathway that may be antagonized by type 2 inflammatory pathways (*45*).

N-cadherin may represent an attractive target for the treatment for both acute and chronic airway obstruction in asthma. Within the airways, N-cadherin expression is limited to the ASM bundles and is up-regulated by TGFβ. In chronic, established asthma, levels of TGFβ are increased, and TGFβ has been previously linked to increased N-cadherin and reduced E-cadherin expression in bronchial epithelial cells (*46*). TGFβ levels are higher in sputum from patients with severe AHR than in those without (*22*, *47*).

Here, we showed that a nontoxic, reversible antagonist of N-cadherin–mediated intercellular interactions (ADH-1) reduced AHR in allergen-challenged mice and prevented bronchoconstriction ex vivo. ADH-1 also elicited bronchodilation in constricted airways, even those desensitized to β-agonists, suggesting that rapidly acting inhaled N-cadherin antagonists might be developed for the treatment of acute airway obstruction in patients with severe bronchoconstriction. ADH-1 is well tolerated by patients with few side effects in clinical trials for solid tumors (*48*). Since ADH-1’s bronchoprotective benefits are independent of inflammation, it may also have benefits for other obstructive lung diseases (chronic obstructive pulmonary disease and viral-associated bronchiolitis).

To constrict the bronchial lumen, ASM cells must contract as a collective. Although it is recognized that multicellular migration within tissues requires long-distance, cooperative transmission of intercellular forces (*49*), the mechanisms underlying ASM cell-cell contraction are not as well understood. In contrast to endothelial cells, which form adherens junction–mediated single-layer contacts on either side, ASM cells form intercalated contacts with multiple cells at once, in all directions (*50*). Because of this unique organizational structure, contraction of the ASM bundle requires complex spatiotemporal remodeling. Using traction force microscopy, we demonstrated that regions of high traction force (“hotspots”) spanned a greater area, coordinated among multiple cells, under control conditions than when exposed to ADH-1. Hotspots comprised approximately 72% of the overall imaged area in vehicle-treated cells compared to 40% in cells treated with ADH-1 for 24 hours.

ADH-1 inhibited ASM contraction by interfering with actin cytoskeletal reorganization independent of canonical pathways leading to ASM shortening (Ca^2+^ flux and MLC phosphorylation). F-actin was primarily cortical (perimembranous) in ADH-1–pretreated cells. This static and poorly coordinated force terrain is expected to impair aligned F-actin stress fiber formation within individual cells and in turn impede cell contraction and synchronized intercellular force transmission in response to a spasmogen (*51*).

Our mechanistic studies and published work suggest that N-cadherin–mediated mechanotransduction depends on interactions with catenin family scaffold proteins, which link N-cadherin to the actin cytoskeleton. While previous in vitro studies suggested that N-cadherin promotes ASM contraction through β-catenin, this interaction has no impact on actin polymerization (*39*, *40*). Here, we find that blockade of N-cadherin homotypic interactions in ASM cells strongly impairs actin stress fiber formation and interactions with δ-catenin, a molecule that regulates RhoA activity (*52*). Active (GTP-bound) RhoA promotes ASM contraction through multiple mechanisms including phosphorylation of type 2 myosin and association with accessory proteins such as S100A4, which in turn facilitates cortical actin-myosin interactions, adhesome assembly, and actin stress fiber formation (*53*).

N-cadherin could also affect actin-independent mechanosignaling to sustain contraction. Bronchi/bronchioles perceive contractile agonist concentrations in concert by generating synchronized Ca^2+^ oscillations propagated around their perimeter to constrict the airway. Agonist-induced Ca^2+^ oscillation frequency increases in proportion to the degree of bronchoconstriction and depends strictly on ECM stiffness and force transmission between adjacent ASM cells rather than intercellular Ca^2+^ diffusion or gap junction–mediated transport (*54*). Although we found that ADH-1 did not reduce the immediate intracellular Ca^2+^ flux elicited by agonist, it may regulate Ca^2+^ oscillations over longer times.

Our study has several limitations. ADH-1’s capacity to overcome β2-adrenergic receptor desensitization needs to be studied further using longer (>12 hours) formoterol pre-exposures (*55*). Although partial N-cadherin deficiency in ASM nearly abolished AHR in allergen-challenged mice, heterozygous gene knockout mice often display compensatory effects that have been attributed to several mechanisms including functional gene redundancy or transcriptional adaptation (*56*). N-cadherin is also expressed in other lung structural cells including epithelial cells (*57*) and fibroblasts (*58*). Although compensatory changes in N-cadherin expression in these cell types in *Cdh2^fl/+^-SMAA-Cre* mice could indirectly affect ASM contractility, the absence of major alterations in lung inflammation and remodeling argue against a major role. Moreover, our RNA sequencing results suggest that a vastly altered transcriptional landscape does not underlie the lack of AHR in these mice.

To address these potential confounding factors experimentally, we confirmed the hypo-contractile ASM phenotype in *Cdh2^fl/+^-SMAA-Cre* mice by depleting N-cadherin expression acutely in two separate models. Treatment of PCLS from adult *Cdh2^fl/+^* mice with cell permeant tat-Cre to induce *Cdh2* gene excision nearly abolished bronchoconstriction. Traction in HASM cells depleted of N-cadherin by means of *Cdh2*-specific siRNA was significantly reduced relative to controls. Future studies of allergen induced AHR in adult mice with inducible, complete N-cadherin knockout might be needed to fully understand the mechanisms driving the hypercontractile phenotype.

ADH-1 had less robust effects on AHR in mice than did *Cdh2* haploinsufficiency, which may relate to compound bioavailability. ADH-1’s half-life in plasma is short (~1.5 to 2 hours) (*59*), and poor solubility precluded direct inoculation to the respiratory mucosa at doses comparable to those used systemically. Moreover, ADH-1’s inhibition of airway contraction in PCLS was lost rapidly after removal of the drug, suggesting prompt re-formation of N-cadherin–mediated cell junctions in the absence of compound. LCRF-006, a small-molecule mimetic of ADH-1, has shown promise for the treatment of hematological malignancies in preclinical models (*60*). Newer generation N-cadherin antagonists with increased potency or bioavailability must be developed for inhalational administration to circumvent the limitations of ADH-1. Further characterization of N-cadherin expression and regulation in various obstructive lung diseases will be important to determine the most suitable applications of N-cadherin–targeted therapeutics in constricted airways.

## MATERIALS AND METHODS

### Study design

The main objective of this study was to elucidate the functions of N-cadherin on ASM contraction at homeostasis and in asthma models. We studied a genetic model (selective *Cdh2* haploinsufficiency in ASM) or effects of a pharmacological antagonist (ADH-1) on HASM monolayer or PCLS contraction ex vivo and AHR induced by fungal allergen challenge. Sample size justification was derived from previous animal studies that were sufficiently powered to detect differences in lung resistance (*24*). Mice were randomly assigned to treatment with vehicle or ADH-1 before the start of each study.

### Cell lines

HASM cells were extracted postmortem from tracheas of de-identified donors [International Institute for the Advancement of Medicine (Edison, NJ) or the National Disease Research Interchange (Philadelphia, PA)] as described previously (*61*). Cells were cultured in Ham’s F-12 medium supplemented with l-glutamine and 10% fetal bovine serum at 37°C in 5% CO_2_.

### Reagents

Recombinant tat-Cre recombinase, TGFβ, EGF, ADH-1, formoterol, MCh, CCh, and histamine were from Sigma-Aldrich. Recombinant human IL-4, IL-13, TSLP, IL-33, and PDGFβ were purchased from R&D Systems.

### Mice

*Cdh2^fl/+^* mice were obtained from The Jackson Laboratoryand backcrossed with Balb/cJ mice for four generations. SMAA-Cre mice were provided by F. Finkelman (University of Cincinnati School of Medicine). All mice were bred and maintained under pathogen-free conditions at an American Association for the Accreditation of Laboratory Animal Care accredited animal facility at the NIAID and housed in accordance with the procedures outlined in the Guide for the Care and Use of Laboratory Animals under an animal study proposal (LAD3E) approved by the NIAID Animal Care and Use Committee.

### Allergic airway inflammation model

Eight- to 12-week-old female mice were sensitized with a mixture of one part alum and one part PBS containing *Af* extract (25 μg of protein, Hollister Stier Allergy), by intraperitoneal injection on days 0 and 7. One to 2 weeks later, mice were then challenged intranasally with either PBS or *Af* (20 μg) daily for three consecutive days (fig. S1). Twenty-four hours after the final challenge, mice were euthanized following analysis of lung resistance. BALF was collected by injection and collection of PBS/1 mM EDTA (1 ml) through a tracheal cannula. Red blood cells were lysed with ACK lysis buffer and clarified BALF supernatants frozen and stored at −80°C. Cell pellets were resuspended in PBS/EDTA counted by hemocytometry and dispersed on glass microscope slides by cytospin. Diff-Quick stained slides were used to determine cell composition by microscopy (*n* = 300 cells per slide). Left lungs were fixed in 10% neutral buffered formalin for generation of paraffin-embedded sections. Sections were stained with hematoxylin and eosin, PAS, or Masson Trichrome. The severity of lung inflammation was evaluated in a blinded fashion using a scoring system as previously described (*29*). In brief, sections were graded on a scale from 0 to 4 based on the degree of inflammatory cell infiltrate: 0, no inflammation; 1, scattered inflammatory cells; 2, a ring of inflammatory cells one cell layer deep; 3, a ring of inflammatory cells two to four cells deep; and 4, a ring of inflammatory cells of >4 cells deep. PAS and Trichrome staining were quantified using the appropriate color deconvolution plugin in ImageJ as described previously (*62*). Cytokines in BALF supernatants were analyzed using a customized MultiPlex bead array (BioRad) (*24*).

### Lung resistance measurements

Mice were anesthetized with a mixture of ketamine (100 mg/kg) and xylazine (10 mg/kg) i.p. The trachea was dissected and cannulated with a 20-gauge catheter. Mice were then paralyzed with vecuronium bromide (200 mg via intraperitoneal injection) and mechanically ventilated using the FlexiVent FX1 respirator (Scireq). Lung resistance was measured by the pulse oscillometry technique at baseline and after inhalation of increasing doses of MCh as described previously (*24*).

### Analysis of airway contraction in PCLS

PCLS were generated as described previously (*24*) and incubated overnight at 37°C in a tissue culture incubator (5% CO_2_). Intact mouse slices were cryopreserved (*63*). On the day of the experiment, the slices were rapidly thawed, placed in 12- or 24-well plates, and stimulated with MCh (10 μM). Luminal area of the airways in lung slices were compared before and at *t* = 20 min after MCh stimulation using ImageJ. Airway constriction is expressed as the percentage of the prestimulation luminal area.

### RNA isolation and quantitative RT-PCR

RNA was extracted using the RNeasy kit (Qiagen) according to the manufacturer’s instructions. Total RNA (500 ng) was reverse transcribed into cDNA using SuperScript IV VILO Master Mix (Thermo Fisher Scientific). TaqMan probes (Thermo Fisher Scientific) were as follows: *Cdh2*, Mm01162497_m1; and *Actb*, Mm02619580_g1.

### RNA sequencing

CTRL and *Cdh2^fl/+^-SMAA Cre* were sensitized and challenged with *Af* and extraction of lung RNA as above. RNA samples were reverse transcribed to cDNA and libraries constructed using the TruSeq DNA Library Preparation Kit (Illumina). Samples were sequenced on the Illumina platform (40 M reads per sample, Beckman-Coulter Genomics). Raw fastq files were trimmed for quality and adapter contamination using Trimmomatic 0.38. Trimmed reads were mapped to a reference genome (UCSC mm10) with HISAT2 version 2.1.0. StringTie version 2.1.3b was used for transcript assembly. The expression profile was calculated for each sample and transcript/gene as a read count, fragment per kilobase of transcript per million mapped reads, and transcripts per kilobase million. Genes with fewer than 10 counts in at least two samples were removed from the dataset, across a total of eight samples. Differential expression analysis was performed using the DESeq2 package (version 1.42.1) in R (version 4.3.1). Normalization was performed using DESeq2’s default size factor method to correct for sequencing depth. The design formula “~ condition” was used to model the effects of the experimental conditions (control versus knockdown). Differentially expressed genes were identified by fitting a negative binomial generalized linear model, and statistical significance was determined using the Wald test. Multiple-testing correction was applied using the Benjamini-Hochberg method to control the false discovery rate (FDR). Log2 fold change estimates were refined using the “lfcShrink” function with the “apeglm” method for more accurate effect sizes, minimizing the impact of extreme values. Genes with an adjusted *P* value (FDR) < 0.05 and an absolute log2 fold change (log2fc) > 0.2 were considered significantly differentially expressed. All analysis was conducted in R (version 4.3.1), and visualizations were created using ggplot2 and heatmap packages.

### Immunoblotting, immunoprecipitation, immunofluorescence, and image analysis

Lysates were prepared from HASM cells using 1× radioimmunoprecipitation (RIPA) lysis buffer (Millipore/Sigma) containing protease (c0mplete) and phosphatase inhibitor (PhosSTOP) inhibitor cocktails (Roche) and clarified by centrifugation at 15,000 rpm for 10 min at 4°C. Samples were boiled with SDS sample buffer at 95°C, electrophoresed on 4 to 12% tris-glycine gels, and transferred to nitrocellulose membranes. Primary antibody staining was detected using near-infrared conjugated secondary antibodies and quantified with the LiCor Odyssey Imaging System and Image Studio software (LiCor Biosciences). HASM cell lysates were immunoprecipitated in buffer containing 50 mM tris (pH 7.5), 150 mM NaCl, 2 mM MgCl_2_, 1 mm EDTA, and 0.5% Triton X-100 using N-cadherin–coupled agarose beads overnight at 4°C followed by three washes with immunoprecipitation buffer. For immunofluorescent staining, cells were plated in Chamberwell slides (Nunc), fixed in 4% paraformaldehyde, and permeabilized with PBS containing 0.2% (v/v) Triton X-100. Cells were then blocked in 2% (w/v) bovine serum albumin in PBS containing 5% goat serum. Cells were then incubated primary antibodies or phalloidin and then with AlexaFluor-conjugated goat anti-mouse or goat anti-rabbit secondary antibodies as outlined in data file S2. Slides were mounted on coverglass with ProLong Gold antifade reagent with 4′,6-diamidino-2-phenylindole. Images were obtained at 63× original magnification using a Leica SP8 confocal microscope and analyzed using ImageJ. Actin fiber anisotropy was analyzed with the FibrilTool plugin. We used Imaris 10.0.0 (BitPlane) and ImageJ to analyze lung sections. Superimposed layers of RGB images were generated by Imaris and converted to 8-bit grayscale images for each channel using ImageJ. The thickness of the ASM layer was measured by SMAA immunostaining essentially as described previously (*64*) The F-actin^+^ area was divided by the total SMAA^+^ area for each airway.

### Cell viability assay

Viability, cytotoxicity, and apoptosis of HASM cells were evaluated using the ApoTox Glo assay (Promega) according to the manufacturer’s instructions.

### Ca^2+^ measurements

HASM cells were plated in 96-well black-walled plates (1 × 10^5^ cells per well). Ca^2+^ Fluo-6 indicator (FLIPR Calcium 6 assay kit, Molecular Devices) and probenecid (1 mM) were added to each well containing serum-free medium and analyzed using a FlexStation III instrument (Molecular Devices) after addition of agonist as described previously (*24*).

### RhoA activation assay

RhoA-GTP in cell lysates was detected by pulldown with GST-Rhotekin–coupled beads using the Active Rho Detection Kit (Cell Signal Technology, catalog no. 8820) as per the manufacturer’s instructions.

### Traction force microscopy

HASM cells were plated upon deformable substrates (Young’s modulus, 0.3 kPa) with embedded fluorescent beads prepared in a standard six-well plate and treated with either vehicle or ADH-1 for 24 hours. The six-well plate was mounted within a heated chamber (37°C) upon an automated computer-controlled motorized stage and imaged at 10× magnification using a monochrome camera (DFC365 FX, Leica) affixed to an inverted microscope (DMI 6000B; Leica). We acquired fluorescent images of microspheres (diameter, ~500 nm) embedded in the elastic substrate immediately underneath the cells at baseline, after histamine treatment, and after cell detachment with trypsin (reference null-force image). By comparing the reference fluorescent images with the corresponding images at baseline and after treatment, we obtained a time series of bead displacement and hence substrate deformation fields (resolution, ∼15 μm). Using the measured substrate deformation, the predefined substrate modulus, and thickness, we calculated traction force maps and the corresponding strain energy (i.e., the energy that is imparted to the substrate by the contractile cells, in pJ, representing the total cell contraction) over a 882 μm by 882 μm area on a well-by-well basis, using the approach of Fourier transform traction cytometry (*33*) modified for cell monolayers (*34*). From each traction map, we also computed the probability distribution function of the traction data to quantify the force distribution (*34*).

### Statistical analysis

Data were analyzed using GraphPad Prism 9.0 and expressed as means ± SEM of *n* = 3 or more biological replicates. Unpaired, two-tailed Student’s *t* test was used for comparison of two groups and one- or two-way analysis of variance (ANOVA) for multiple groups. Nonparametric analyses were used for nonnormally distributed data. *P* values <0.05 were considered statistically significant.
