# Supplementary material for: N-cadherin antagonism is bronchoprotective in severe asthma models
Source: Sci Adv. 2024 Nov 29;10(48):eadp8872. doi: 10.1126/sciadv.adp8872 (PMC11606448; doi:10.1126/sciadv.adp8872)
Supplement: Supplementary file 1 — Figs. S1 to S9 Table S1 Legend for data files S1 and S2 [file sciadv.adp8872_sm.pdf]

Science Advances
AAAS

Supplementary Materials for

N-cadherin antagonism is bronchoprotective in severe asthma models

Nicolas L. Pereira *et al.*

Corresponding author: Kirk M. Druey, kdruey@niaid.nih.gov

Sci. Adv. **10**, eadp8872 (2024)
DOI: 10.1126/sciadv.adp8872

The PDF file includes:

Figs. S1 to S9
Table S1
Legend for data files S1 and S2

Other Supplementary Material for this manuscript includes the following:

Data files S1 and S2

Fig S1

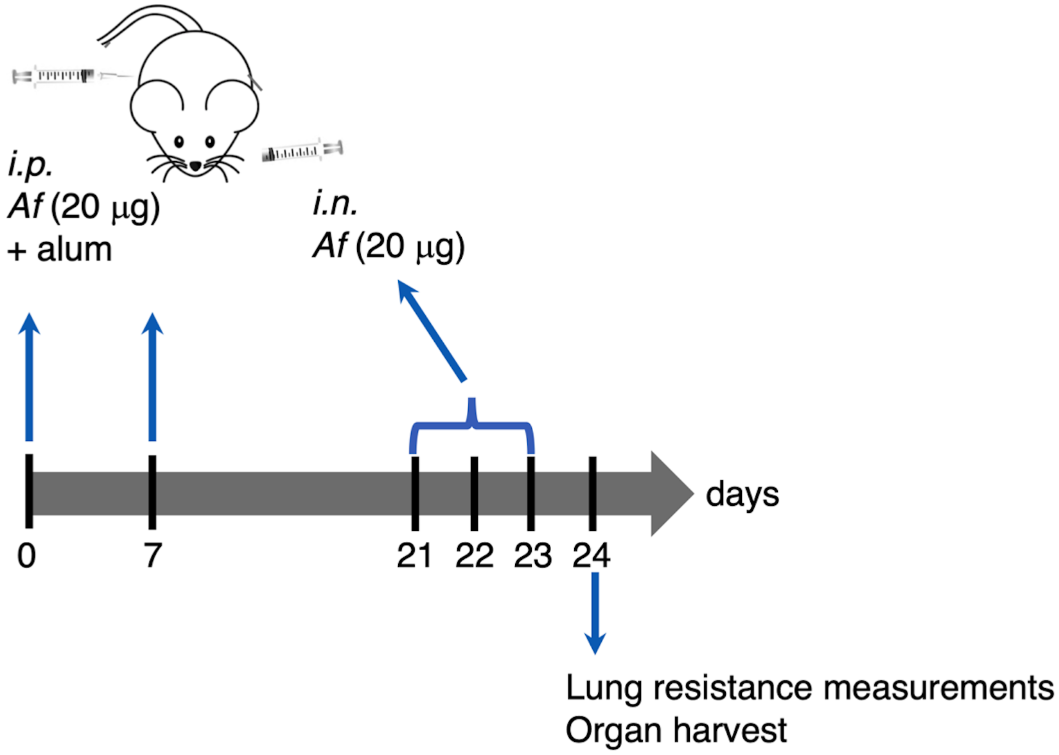

Figure S1 Schematic of the allergen sensitization and challenge model. Mice were sensitized with two doses of *Af* extract administered intraperitoneally (i.p.). Two weeks later, mice were challenged intranasally (i.n.) for 3 consecutive days with *Af*. 24 hours after the last challenge, lung resistance was assessed in live, mechanically ventilated mice by plethysmography. Mice were then sacrificed, followed by collection of BALF and organ harvest.

Fig S2

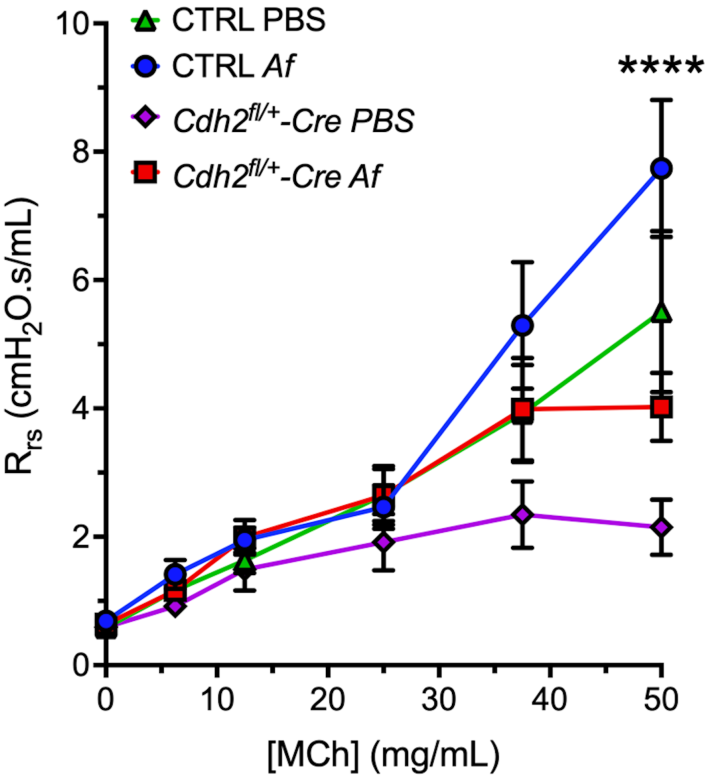

Figure S2 Total lung resistance in N-cadherin deficient mice. Respiratory system resistance (R_{rs}) in naïve or *Af*-challenged mice. Means ± SEM from n=10-15 mice/group. ****P<0.0001 (CTRL vs. *Cdh2*$^{fl/+}$-*SMAA Cre*, PBS or *Af*-treated), two-way ANOVA, Tukey multiple comparisons.

Fig S3

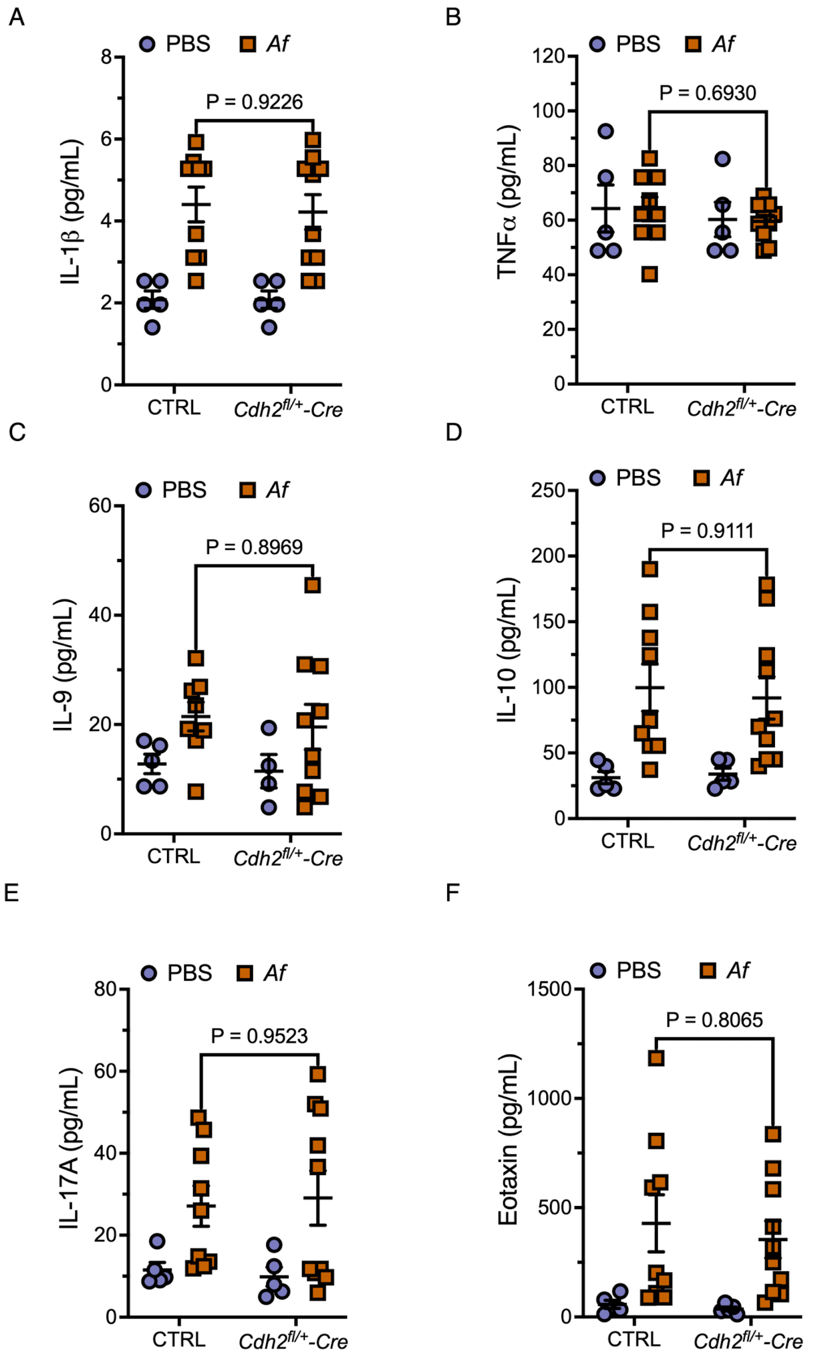

Figure S3 Asthma-related cytokines in BALF. Levels of IL-1β (**A**), TNFα (**B**), IL-9 (**C**), IL-10 (**D**), IL-17A (**E**), or eotaxin (**F**) in BALF from *Cdh2$^{fl/+}$-SMAA Cre* or control mice. Means ± SEM from n=5-10 mice/group. *P* values were determined by two-way ANOVA with Sidak multiple comparisons.

Fig S4

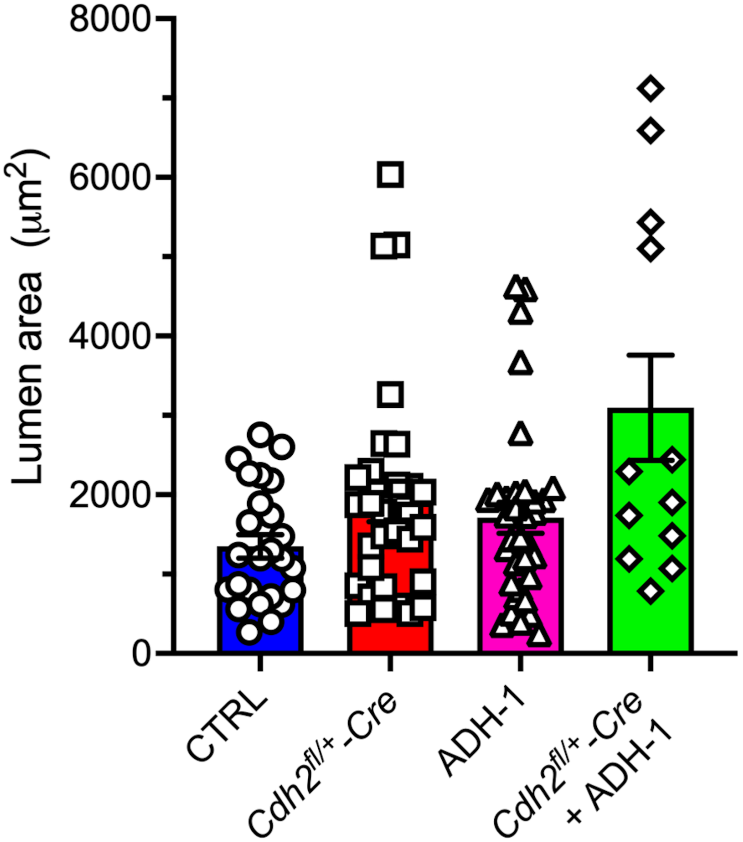

Figure S4 N-cadherin deficiency or ADH-1 effect on baseline airway luminal areas in PCLS. Areas determined from brightfield images using Image J. Means ± SEM from n=13-34 airways/group.

Fig S5

A

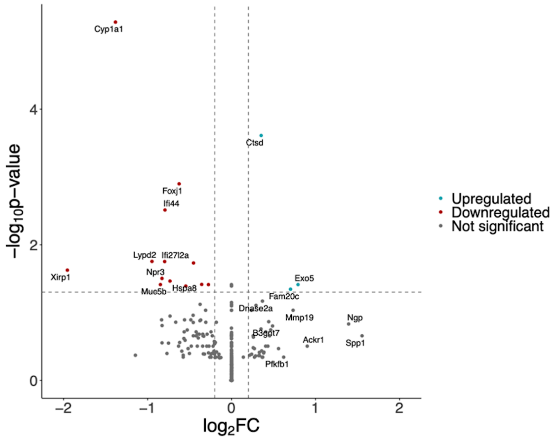

B

#category	term ID	term description	observed gene count	background gene count	strength	false discovery rate	matching proteins in network (IDs)
**Monarch**	MP:0005387	Immune system phenotype	14	3467	0.64	0.00085	0090.ENSMUSP00000003910,10090.ENSMUSP00000026411,10090.ENSMUSP010000026972,10090.ENSMUSP00000029671,10090.ENSMUSP00000035061,10090.ENSMUSP00000038351,10090.ENSMUSP00000045134,10090.ENSMUSP00000054698,10090.ENSMUSP00000056836,10090.ENSMUSP00000066737,10090.ENSMUSP00000084043,10090.ENSMUSP00000121203,10090.ENSMUSP00000128276,10090.ENSMUSP00000150277
**Monarch**	MP:0008469	Abnormal protein level	9	1450	0.83	0.0095	10090.ENSMUSP00000003910,10090.ENSMUSP00000026411,10090.ENSMUSP00000026972,10090.ENSMUSP00000045134,10090.ENSMUSP00000054698,10090.ENSMUSP00000066737,10090.ENSMUSP00000084043,10090.ENSMUSP00000121203,10090.ENSMUSP00000150277
**Monarch**	MP:0005416	Abnormal circulating protein level	8	1316	0.82	0.0334	10090.ENSMUSP00000003910,10090.ENSMUSP00000026972,10090.ENSMUSP00000045134,10090.ENSMUSP00000054698,10090.ENSMUSP00000066737,10090.ENSMUSP00000084043,10090.ENSMUSP00000121203,10090.ENSMUSP00000150277
**Compartments**	GOCC:0005615	Extracellular space	7	765	1	0.0076	10090.ENSMUSP00000015800,10090.ENSMUSP00000026972,10090.ENSMUSP00000035061,10090.ENSMUSP00000056836,10090.ENSMUSP00000084043,10090.ENSMUSP00000121203,10090.ENSMUSP00000128276

Figure S5 Gene expression patterns in control vs. smooth muscle specific *Cdh2* haploinsufficient mice. (**A**) Volcano plot of the most significantly differently expressed genes in *Af*-challenged *Cdh2*$^{fl/+}$-*Cre* or control mice determined by RNA-Seq (n=4 mice/group). Dashed line designates genes with a significant FDR (adjusted *P* value). (**B**) Pathways enriched in differentially expressed gene sets together with Benjamini–Hochberg-corrected *P* values.

Fig S6

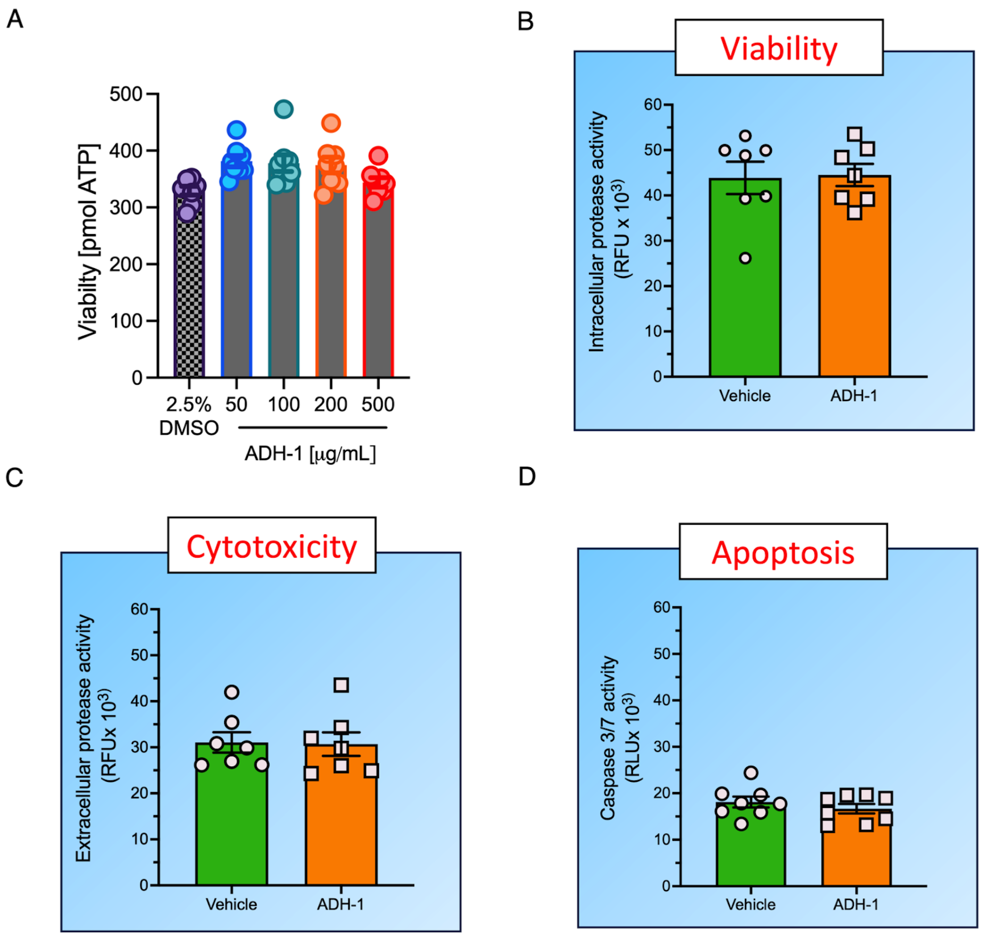

Figure S6 ADH-1 does not induce cytotoxicity in HASM cells. (**A**) Intracellular ATP levels (viability marker) in HASM cells treated with vehicle (2.5% DMSO) or various concentrations of ADH-1 (in 2.5% DMSO at 500 µg/mL) for 24 hours. (**B-D**) HASM cells treated with ADH-1 (250 µg/mL for 24 hours) followed by assessment of intracellular protease activity (viability marker) (B), extracellular protease activity (a marker of cytotoxicity) or caspase 3/7 activity (apoptosis marker) by multiplexed assay.

Fig S7

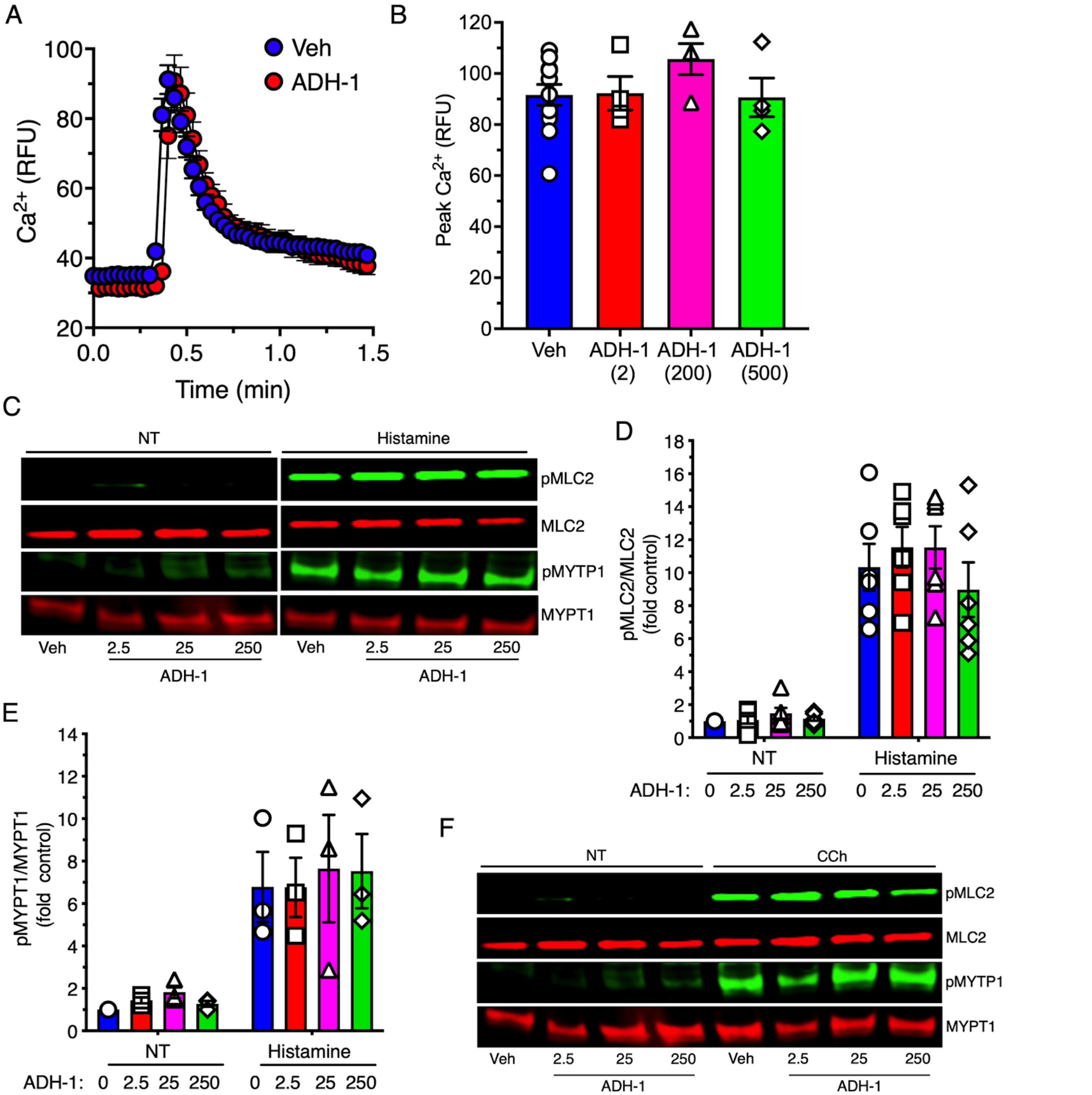

Figure S7 ADH-1 inhibits HASM contraction independent of canonical excitation-contraction pathways. (**A-B**) Intracellular Ca^{2+} (relative fluorescence units, RFU) over time (A) and peak amounts (B) in HASM cells pre-treated with vehicle or ADH-1 (μg/mL) for 24 hours and stimulated with histamine (3 μM). Means ± SEM from n=4-12 biological replicates/group. (**C-F**) HASM cells pretreated with vehicle or ADH-1 (μg/mL) for 24 hours and stimulated with histamine (1 μM, C) or carbachol (CCh) (20 μM) (F) for 10 minutes. Representative blots (C, F) probed with antibodies against phosphorylated or total myosin light chain 2 (MLC2) or myosin light chain phosphatase 1 (MYPT1). Quantified data (means ± SEM) from n=3-6 donors/group are presented in panels D-E.

Fig S8

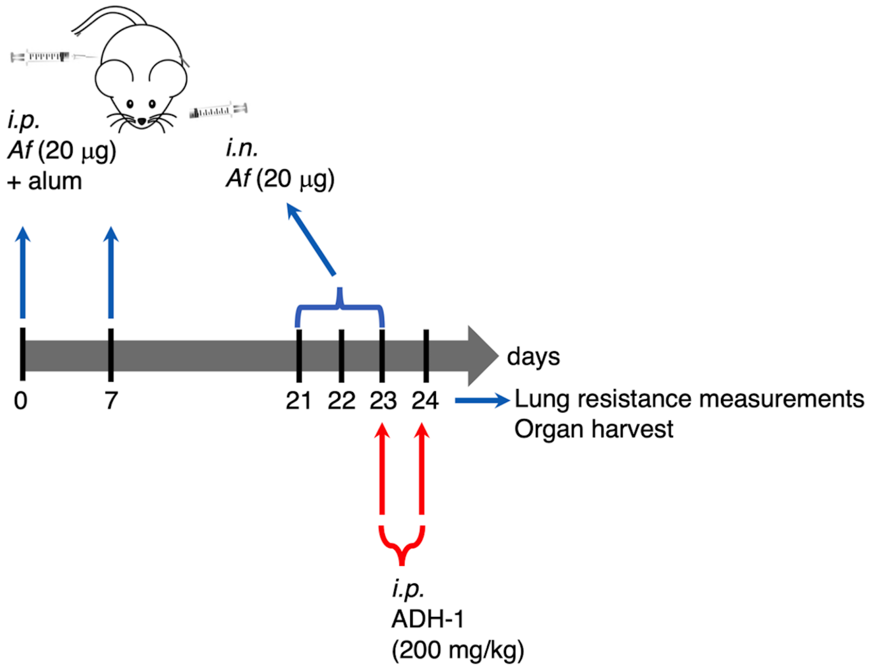

Figure S8 Schematic of ADH-1 treatment in the experimental asthma model. Mice were sensitized with two doses of *Af* extract administered intraperitoneally (i.p.). Two weeks later, mice were challenged intranasally (i.n.) for three consecutive days with *Af*. Vehicle or ADH-1 (200 mg/kg) was then administered i.p. 18 hours and 30 minutes prior to plethysmography.

Fig S9

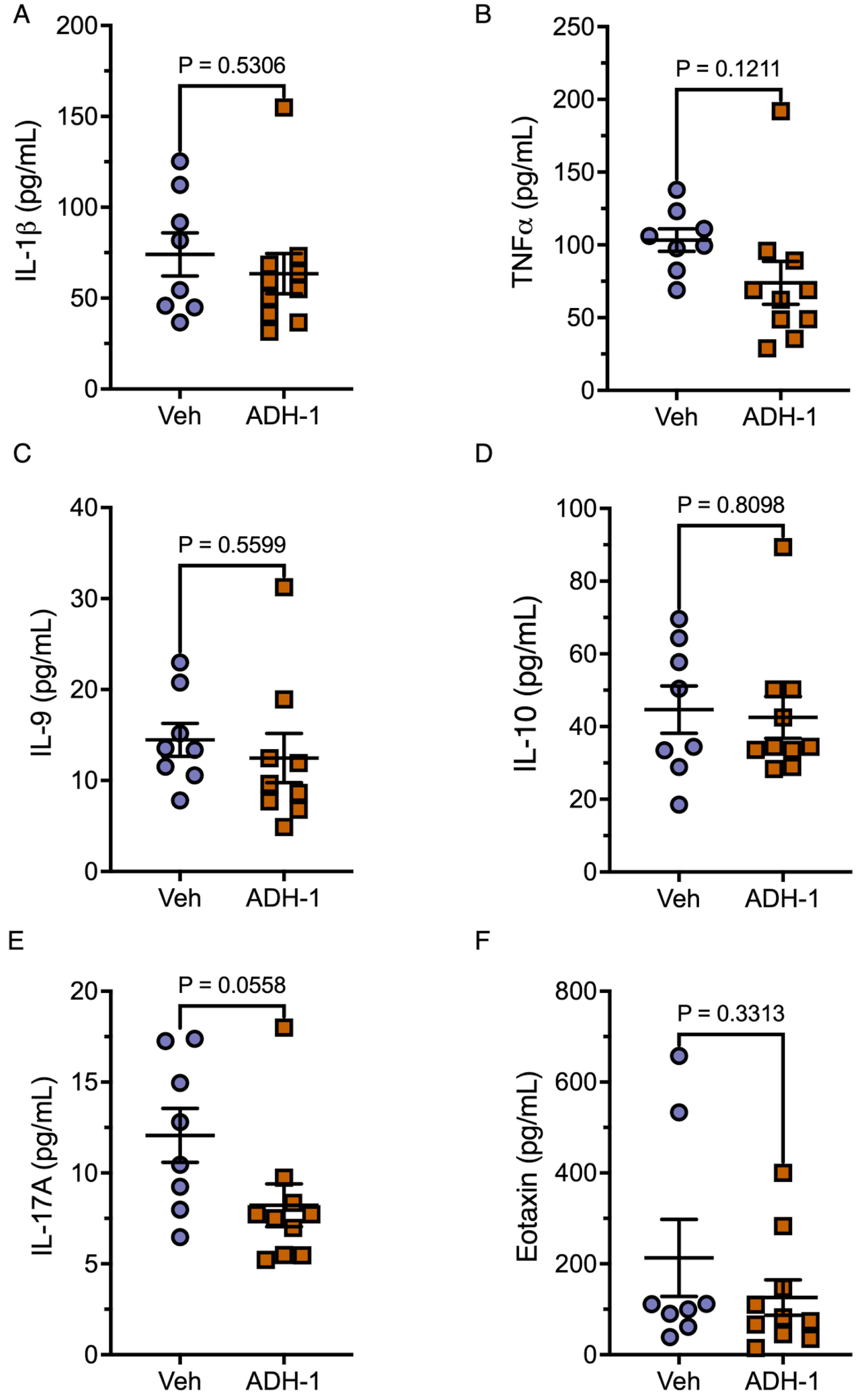

Figure S9 Effect of ADH-1 on asthma-related cytokines in allergen challenged mice. Levels of IL-1β (**A**), TNFα (**B**), IL-9 (**C**), IL-10 (**D**), IL-17A (**E**), or eotaxin (**F**) in BALF from vehicle or ADH-1-treated mice. Means ± SEM from n=8-10 mice/group. *P* values were determined by Mann-Whitney *U* test (A) or unpaired *t* test (B-F).

Table S1 Donor Demographics

Parameter	Healthy	Asthma
**N**	8	7
**Age (years)***	24.7 +/- 4	25 +/- 5.3
**Sex ([F/M] (n/%)**	2/8 (25%)	2/7 (28.5%)
**Race [non-white] (n/%)**	3/8 (37.5%)	3/7 (43%)
**BMI***	25.82 +/- 2	25.57 +/- 2.3
**Smoking history (n/%)**	4/8 (50%)	2/7 (28.5%)

* Mean ± SEM

Data File S1 RNA-Seq results. List of differentially expressed genes in *Af*-challenged *Cdh2*$^{fl/+}$-*SMAA Cre* mice compared to controls.

Data File S2 Antibodies used in the study. Dilutions and source information listed.
